# Characterization of *Klebsiella pneumoniae* Isolates Resistant to Cefiderocol from Hospitals and Outpatient Settings in Croatia

**DOI:** 10.3390/antibiotics14020154

**Published:** 2025-02-04

**Authors:** Branka Bedenić, Josefa Luxner, Gernot Zarfel, Ana Benčić, Sanda Sardelić, Maja Anušić, Jasmina Vraneš, Verena Dobretzberger, Ivan Barišić, Andrea Grisold

**Affiliations:** 1 Biomedical Research Center-BIMIS, University of Zagreb School of Medicine, 10000 Zagreb, Croatia; 2 Clinical Department for Clinical Microbiology and Infection Control and Prevention, University Hospital Centre Zagreb, 10000 Zagreb, Croatia; 3 Diagnostic and Research Institute of Hygiene, Microbiology and Environmental Medicine, Medical University of Graz, 8036 Graz, Austria; josefa.luxner@medunigraz.at (J.L.); gernot.zarfel@medunigraz.at (G.Z.); andrea.grisold@medunigraz.at (A.G.); 4 Department of Gynecology and Obstetrics, University Hospital Centre Sestre Milosrdnice, 10000 Zagreb, Croatia; anabeni62@gmail.com; 5 Department of Microbiology and Parasitology, University Hospital of Split, 21000 Split, Croatia; sandasard@gmail.com; 6 Department of Clinical Microbiology, Dr. Andrija Štampar Teaching Institute of Public Health Zagreb, 10000 Zagreb, Croatia; manusic.40@gmail.com (M.A.); jasmina.vranes@stampar.hr (J.V.); 7 Department of Medical Microbiology and Parasitology, University of Zagreb School of Medicine, 10000 Zagreb, Croatia; 8 Department of Molecular Diagnostics, Austrian Institute for Technology, 1210 Vienna, Austria; verena.dobretzberger@ait.ac.at (V.D.); ivan.barisic@ait.ac.at (I.B.)

**Keywords:** *Klebsiella pneumoniae*, cefiderocol, OXA-48, KPC, metallo-β-lactamase

## Abstract

Background/Objectives: We conducted this study to evaluate the genotypic and phenotypic profiles of carbapenem-resistant *Klebsiella pneumoniae* (CRKP) isolates, exhibiting resistance to cefiderocol (FDC), focusing on antibiotic susceptibility, β-lactamase production, the genetic environment of *bla*_CARB_ and *bla*_ESBL_ genes and molecular epidemiology. FDC is now a last-line antibiotic for severe infections due to CRKP. Methods: Susceptibility to a wide range of antibiotics was determined by the disk diffusion and broth microdilution method. Carbapenemases were screened by a modified Hodge test while carbapenem hydrolysis was investigated using mCIM and eCIM tests. The screening for β-lactamase and fluoroquinolone cluster resistance genes was carried out by PCR. Plasmids were characterized by PCR-based replicon typing (PBRT). An inter-array genotyping CarbaResist test and whole genome sequencing (WGS) were applied on selected isolates. Results: All of the 31 isolates studied exhibited high-level resistance to amoxicillin–clavulanate, piperacillin–tazobactam, cefuroxime, expanded-spectrum cephalosporins (ESC), cefepime, ceftolozan–tazobactam and ciprofloxacin and the majority to gentamicin, and amikacin. Colistin and ceftazidime–avibactam preserved activity against 71% and 87% of the isolates, respectively. The combined disk method with clavulanic acid was positive in all but one isolate, indicating the production of an ESBL. Twenty-eight isolates carried one single carbapenemase-encoding gene, whereas three harbored double *bla*_CARB_ genes. Among the studied isolates, 61% carried *bla*_OXA-48_, 29% *bla*_KPC_ and 12.9% *bla*_NDM_ genes. The inter-array genotyping CarbaResist test and WGS identified additional aminoglycoside-, sulphonamide- and trimethoprim-resistance genes. Conclusion: To our knowledge, this is the first study on FDC resistance in Croatia. The diffusion of FDC-resistant isolates was detected in both hospital and outpatient settings, emphasizing the need for a “One Health” approach.

## 1. Introduction

Antibiotic resistance is a natural characteristic of microorganisms, existing before the use of antibiotics [1]. The indiscriminate use of antimicrobials in clinical practice has resulted in selective pressure, which is responsible for the spread of antibiotic resistance [2].

*Klebsiella pneumoniae* is one of the most important hospital pathogens due to its capability to cause severe infections like pneumonia in ventilated patients, bloodstream infections, urinary tract and wound infections [3,4]. *K. pneumoniae* isolates of major concerns are those harboring extended-spectrum β-lactamases (ESBLs), plasmid-mediated AmpC β-lactamases (p-Amp-C) and carbapenemases leading to a multidrug-resistant (MDR) phenotype [5,6,7]. Resistance to carbapenems is caused by enzymatic inactivation mediated by carbapenemases (KPC, IMP, VIM, NDM, OXA-48) spreading via mobile genetic elements, permeability alterations caused by the loss of OmpK35 and OmpK36 and hyperexpression of efflux systems [8]. OXA-48 belonging to class D or carbapenem-hydrolyzing oxacillinases (CHDL) are now dominant in the majority of European countries [8]. The emergence and spread of MDR strains severely limit therapeutic options, which poses a global public health threat. Among carbapenemases, Ambler class B or metallo-β-lactamaes (MBLs) represent the greatest challenge to clinicians due to the limited therapeutic options [8]. Colistin is one of the antibiotics, in addition to beta-lactam-inhibitor combinations, used in the treatment of infections associated with carbapenem-resistant *K. pneumoniae* (CRKP) [9]. Colistin resistance emerged among ESBL positive *K. pneumoniae* and CRKP leading to the extensively drug-resistant (XDR) phenotype [9]. This limits therapeutic options, resulting in difficult-to-treat or untreatable infections [10,11]. Aminoglycoside resistance in CRKP is mediated by the acquisition of *aac*, *aad* and *aph* genes, often encoded on the same plasmid as carbapenemase-coding genes. New β-lactam-inhibitor combinations such as ceftolozane–tazobactam, ceftazidime–avibactam and imipenem–cilastatin–relebactam are now last resort antibiotics for infections due to CRKP. However, they exert poor activity against isolates producing class B carbapenemases or MBLs [12]. New-generation cephalosporins are shown to possess excellent activity against CRKP, producing all types of carbapenemases (KPC, IMP, VIM, OXA-48) [12].

Cefiderocol (FDC) is the first-in-class catechol–siderophore–cephalosporin approved in the European Union (EU), with potent activity against carbapenemase-producing Enterobacterales, *Pseudomonas aeruginosa* and *Acinetobacter baumannii* [12]. FDC binds to penicillin-binding proteins (PBP) to block the final stage of peptidoglycan synthesis [13]. Its activity is not compromised by upregulation of efflux pumps or porin alteration [13]. FDC is used for the treatment of complicated urinary tract infections, hospital-acquired bacterial pneumonia and ventilator-associated pneumonia [14].

However, there have been increasing reports indicating correlations between the production of carbapenemases and reduced susceptibility to FDC [15]. The activity of FDC is decreased in the presence of MBLs, particularly NDM, and ESBLs belonging to PER-1 in *Acinetobacter baumannii* and SHV-2 and SHV-5 encountered in Enterobacterales [15]. Other resistance mechanisms to FDC are attributed to iron transport systems defects due to mutations of *cir*A and *fiu* genes, impeding drug entry to the bacterial cells [16,17]. The rate of FDC resistance among Enterobacterales in Europe is only 3% (1.5–6%), but is much higher among carbapenem-resistant Enterobacterales (CRE) and reaches 12.5% (7.3–20%) [18]. FDC is being used in Croatia since a few years ago, and resistance to this compound is still rare. The data from 2023 confirmed an FDC resistance rate of 7% (0–16%) [19] among *K. pneumoniae* in general, but there are no studies on the prevalence of resistance among CRKP. Here, we report the emergence and spread of FDC resistance in CRKP-producing carbapenemases. We conducted this study to evaluate the genotypic and phenotypic profiles of CRKP isolates, exhibiting resistance to FDC, focusing on antibiotic susceptibility, β-lactamase production, the genetic environment of *bla*_CARB_ and *bla*_ESBL_genes and molecular epidemiology.

## 2. Results

### 2.1. Isolates and Patients

The 31 non-copy (one per patient) FDC-resistant isolates were recovered from various clinical specimens, including clinically relevant or surveillance cultures from patients with either a *K. pneumoniae* associated infection or colonization. The majority of isolates originated from the midstream urine (14) and urinary catheter (14). Only one strain was recovered from the blood culture. The remaining two isolates were obtained from surveillance cultures (endotracheal aspirate and rectum swab).

The rate of FDC-resistant isolates against the total number of CRKP isolates in the participating centers in 2023 was 31% in UHS (University Hospital of Split) (374/1208), 19% in PH (Dr Andrija Štampar Teaching Public Health Institute) (79/418) and 0% (0/430) in UHCSM (University Hospital Center Sestre Milosrdnice). Data for 2024 are now available only for UHCSM, which identified 11% (61/552).

### 2.2. Antimicrobial Susceptibility and Phenotypic Tests for β-Lactamases

All of the tested isolates exhibited high-level resistance to amoxicillin–clavulanate, piperacillin–tazobactam, expanded-spectrum cephalosporins or ESC (ceftazidime, cefotaxime, ceftriaxone), cefepime, ceftolozan–tazobactam, sulphamethoxazole–trimethoprim and ciprofloxacin and the majority to gentamicin, imipenem, meropenem (97%, *n* = 30), and amikacin (80%, *n* = 25) (Table 1). The resistance to colistin and ceftazidime–avibactam was rarely detected, with 71% (*n* = 22) and 87% (*n* = 27) of the isolates being susceptible, respectively, as visible in Table 1. The minimum inhibitory concentrations (MIC) values of cephalosporins and ciprofloxacin exceeded 128 mg/L, while the MIC of cefiderocol ranged between 3 and 16 mg/L, as shown in Supplementary Table S1. Two isolates were allocated to an MDR phenotype as they exhibited susceptibility to either carbapenems or aminoglycosides, in addition to colistin and ceftazidime–avibactam. One isolate was PDR (pandrug-resistant), since it was resistant to all available antibiotics for *K. pneumoniae* in Croatia, and the rest were extensively drug-resistant (XDR). MAR (multiple antibiotics resistance) indices varied between 0.75 and 1, with a mean value of 0.88 and median of 0.87. The combined disk method with clavulanic acid as the inhibitor was positive in all but one isolate (97%), while DDST (double-disk synergy test) tested positive in 84% (n = 26) of the isolates, indicating the production of an ESBL. The inhibitor-based test with cloxacillin showed uniformly negative results, confirming the lack of p-AmpC. The modified Hodge test (MHT) exhibited higher sensitivities in detecting carbapenemase production, with only two isolates being false-negative (6.4%) compared to mCIM (modified carbapenem inactivation method), which failed to identify carbapenemase in four isolates (13%). eCIM (EDTA-carbapenem inactivation method) was positive in three out of four MBL positive isolates, while one KPC producer demonstrated a false positive result. OKNV (OXA-48, KPC, NDM, VIM) testing carried out in routine microbiology laboratories revealed OXA-48 in nineteen, KPC in nine, and NDM in four isolates.

### 2.3. Molecular Detection of Resistance Genes

Twenty-eight isolates carried one single carbapenemase-encoding gene whereas three harbored double *bla*_CARB_ genes (Table 1). Among the studied isolates, 61% (n = 19) carried *bla*_OXA-48_, 29% (n = 9) *bla*_KPC_ and 13% (n = 4) *bla*_NDM_ genes, as shown in Table 1. Double carbapenemases were observed in three isolates (two OXA-48 + NDM and one VIM + NDM) (Table 1).

The PCR for *bla*_CTX-M_ genes yielded positive results in 26 out of 30 strains (78%), being phenotypically positive for an ESBL, with all amplicons belonging to phylogenetic group 1. *bla*_CTX-M-15_ was the only allelic variant found. *bla*_OXA-48_ genes were associated with the IS*1999* insertion element upstream of the gene, while IS*Ecp* preceded *bla*_CTX-M_ genes. The other β-lactam-resistance genes identified were *bla*_SHV_-positive, as expected, in all isolates, and *bla*_TEM_-positive in 13 isolates. Allelic variants generated by *bla*_SHV_ were SHV-1 and 11, whereas all *bla*_TEM_ encoded TEM-1. The *qnr*B gene was found in one isolate harboring double carbapenemases.

### 2.4. Detection of Resistance Genes by the Inter-Array Genotyping Kit CarbaResist

Out of four tested representative isolates, two were found positive for double carbapenemase genes, *bla*_VIM_ and *bla*_NDM_ genes, respectively. Two isolates were found to carry the *bla*_OXA-48_ gene (Table 2). A combination of two MBL genes was found in one isolate, whereas one harbored a combination of OXA-48- and NDM-encoding genes, as shown in Table 2. Furthermore, the aminoglycoside-encoding genes *aac*(6′)-Ib, *aad*A1 and *aad*A2 were identified in three strains and *aph*A in one isolate. In addition, the fluoroquinolone resistance-determinant *qnrB* was detected in one isolate, being positive for two MBLs.

All four isolates tested positive for the *sul*1 gene conferring resistance to sulphonamides, with one harboring the *dfrA12* gene, which was responsible for trimethoprim resistance as well. Finally, genes for efflux pumps were present in three isolates.

### 2.5. Whole Genome Sequencing (WGS)

WGS results confirmed the PCR and CarbaResist inter-array genotyping results, but some disconcordances were identified, particularly with β-lactam resistance determinants. WGS failed to identify *bla*_VIM_ genes in isolates 1 and 4, as shown in Table 3. However, it detected *bla*_NDM_ genes in isolates 3 and 4 which were missed by the CarbaResist inter-array genotyping kit and PCR (Table 3). Moreover, genome sequencing detected *bla*_SHV_ genes in two isolates, which were missed by the CarbaResist inter-array genotyping kit. Such loss of resistance is regularly observed in genes after recultivation steps to extract gDNA due to the lack of antibiotic selection pressure in the subcultures [20,21].

There were two allelic variants of *bla*_NDM_ genes: *bla*_NDM-1_ and *bla*_NDM-5_. Aminoglycoside resistance genes were in concordance using both methods, but WGS found an additional *aph* gene in strain 1, not detected by the KitCarbaResist inter-array genotyping test. Trimethoprim-resistance genes were confirmed by both methods, but strain 3 was found to possess the *dfrA12* gene not found by KitCarbaResist inter-array genotyping. Three different allelic variants of *bla*_SHV_ genes were detected: *bla*_SHV-187_, *bla*_SHV-28_, and *bla*_SHV-158_. Regarding *bla*_CTX-M_ genes, there was only one variant present: *bla*_CTX-M-15_.

The *aac*(*6″*)-Ib-cr gene, detected in two isolates, provides combined resistance to aminoglycosides and low-level resistance to norfloxacin and ciprofloxacin. The genes encoding efflux pumps (oqxA and oqxB) were confirmed by both molecular methods.

### 2.6. Plasmid Content

Several plasmid replicons were found including the most frequent IncL, associated with all 19 OXA-48-producing organisms, while IncX3 was found in three out of four NDM-producing organisms. IncN was positive in the strain coharboring VIM and NDM carbapenemases.

### 2.7. Genotyping of the Isolates by Multilocus Sequence Typing (MLST)

One of the strains (1, UG 65815) was found to belong to sequence type (ST) 20 (*gap*A-2, *pho*E-4, *pgi*-229, *inf*B-3, tonB-4, *rpo*B-4, *mdh*-1) while the other (4, 8086-2-24) was classified as ST 4051 (*gap*A-15, *pho*E-1, *pgi*-1, *in*fB-3, *ton*B-31, *rpo*B-1, *mdh*-1). ST for the strain 3 (VG-34989) was retrieved from WGS and was shown to belong to ST15.

## 3. Discussion

Infections caused by MDR bacteria are an alarming problem worldwide, although in recent decades, a great development of new antibiotics was observed in high-income countries. Access to healthcare system is associated with an excessive drug uptake, use of biomaterials, and invasive procedures often complicated with nosocomial infections. The World Health Organization (WHO) declared antimicrobial resistance as one of the greatest threats to global health [22]. According to the WHO, ESBL-positive *K. pneumoniae* and CRKP are listed as critical pathogen and a member of a published list of bacteria for which new antibiotics are urgently needed [22]. The understanding of molecular mechanisms of antimicrobial resistance is important to cope with infections due to these superbugs. Therefore, we aimed to analyze the resistance determinants among these critical pathogens. Other European studies have shown that FDC is superior to novel-β-lactam inhibitor combinations against CRE (88% vs. 66–72% susceptibility rates) [12]. In our study, ceftazidime–avibactam and colistin exhibited activity against the majority of FDC-resistant isolates. The most common species with the problem of FDC resistance worldwide is the *Enterobacter cloacae* complex, which could be due to the overexpression of chromosomal AmpC cephalosporinase. However, the rate of FDC resistance is constantly increasing among *K. pneumoniae* [12]. FDC resistance was associated with an XDR phenotype in the majority of isolates. Only two isolates demonstrated susceptibility to carbapenems and aminoglycosides, respectively, and were categorized as MDR. One PDR isolate was resistant to all available antibiotics active against *K. pneumoniae*, licensed in Croatia. MAR indices were high in all isolates, indicating a very low number of antibiotics exhibiting activity against our isolates, which poses a therapeutic challenge for clinicians.

FDC resistance is usually attributed to multiple resistance mechanisms, including MBL production, ESBL- and AmpC-positivity, iron uptake-related mutation and *ftsl* mutation leading to alteration of PBP3, as reported in previous studies [13]. In our study, resistance was mostly linked to OXA carbapenemases, although the FDC resistance mechanisms were not analyzed in the present study. In contrast to other European studies, our isolates were highly susceptible to ceftazidime–avibactam (87%) and moderately susceptible to colistin (71%, MIC_90_ = 128). Ceftolozane–tazobactam did not exert any activity on our FDC-resistant isolates. Interestingly, FDC-resistant, OXA-48-producing organisms exhibited higher carbapenem MIC values and resistance rates of 100%, compared to previous studies [23,24] in which 18–37% isolates demonstrated resistance to imipenem and 29–47% to meropenem. OXA carbapenemases exert weak carbapenem hydrolysis and high-level resistance is usually due to other resistance mechanisms such as porin loss or upregulation of efflux pumps. Genes for efflux pumps (*oqx*A and *oqx*B) were detected in all but one of the representative tested isolates by the CarbaResist inter-array genotyping kit and WGS. Since the efflux systems pump out antibiotics belonging to different classes, this could explain high-level resistance to other antibiotic categories as well. The strain positive for NDM only was susceptible to imipenem and meropenem, and resistant only to ertapenem. In other European studies, FDC resistance was usually identified in NDM-producing organisms [25]. The Hodge test showed higher sensitivity in detecting carbapenemase activity compared to the CIM test, which did not identify OXA-48 in some of the strains, contrary to other studies on the sensitivity of phenotypic testing for carbapenemases [26]. False negative tests could be attributed to the weak carbapenem hydrolysis exerted by OXA-48. The m/eCIM test was negative in one MBL-producing organism.

On the other hand, DDST failed to detect ESBLs in KPC-producing organisms which were positive in combined disk test with clavulanic acid. This could be explained by inappropriate distances between cephalosporins disks and the central disk with clavulanic acid. Moreover, the production of carbapenemase can antagonize the synergistic effect with clavulanic acid and mask the deformation of the inhibition zone. The combined disk test was shown to have higher sensitivity. Since ESBL-positive, KPC-producing organisms did not harbor *bla*_CTX-M_ genes and *bla*_TEM_ genes generated broad-spectrum TEM-1 β-lactamase, the ESBL positivity was probably linked to some SHV-ESBL variant, but the sequencing was not performed to confirm the statement.

The study documents dissemination of FDC-resistant isolates among *K. pneumoniae* from participating centers in Croatia. The main finding of the study is that FDC resistance was linked to various carbapenemase types and that the isolates harbored a plethora of additional resistance genes as well. This points out to the amazing capacity of *K. pneumoniae* to acquire resistance determinants to almost all available antibiotics, leaving no therapeutic options. FDC resistance in other studies was associated mainly with NDM-MBLs, belonging to ST437, a newly emerging clone [27]. Regional differences in the carbapenemase types were observed in this study. In Split, located in the southern region of Croatia, FDC resistance was most frequently linked to OXA-48 positivity, while in Zagreb, located in central Croatia, the production of KPC in the majority of cases accompanied FDC resistance in the hospital setting. On the other hand, in the outpatient setting, OXA-48 outnumbered all other carbapenemases. NDM as the sole carbapenemase was recorded in only one case, but double carbapenemaes were reported in three cases, two from Split and one from the outpatient setting. Multiple carbapenemases were recorded in the present study in three isolates (two from Split and one from Zagreb), but they were already described in Croatia during the COVID-19 pandemic, with OXA-48 + NDM as the dominant combination [28], but FDC was neither approved for use in Croatia nor tested in routine diagnostic during this period.

The majority of the isolates harbored additional CTX-M β-lactamase. The insertions sequence IS*EcpI* is known to mobilize adjacent sequences, including *bla*_CTX-M_ genes, by using its own left inverted repeat and increases the expression of the gene [29], which might explain very high cephalosporin MICs among OXA-48-producing organisms, in spite of the fact that this type of CHDL does not hydrolyze cephalosporins. The genetic environment of *bla*_OXA-48_ genes was consistent with previous work [23].

Fluoroquinolone resistance was attributed to a plasmid-mediated *qnr*B gene in one and to *aac*(6″)-Ib-cr in two isolates, whereas in other isolates, it was probably attributed to mutations of *gyr*A and *par*C genes which is consistent with very high MICs of ciprofloxacin exceeding 128 mg/L. These mutations cannot be identified with WGS or the CarbaResist inter-array genotyping kit. The fluoroquinolone-resistance genes were searched for because they often reside on the same plasmid-encoding ESBLs and carbapenemases. A strong correlation between resistance genes and phenotypic resistance patterns was proved, indicating the expression of the detected resistance genes. The numerous aminoglycoside resistance genes were correlated with the very high MICs of gentamicin and amikacin. However, in one of the strains susceptible to gentamicin and amikacin, *aac*(3), *aac(*6) and aph genes were recorded using a molecular approach, but obviously not phenotypically expressed.

There was no difference in resistance phenotype between hospital and outpatient isolates. Both settings had isolates with the MDR or XDR phenotype, but in the outpatient setting, we did not have any data available on previous hospitalization or residence in the long-term facilities, which are a known source of resistant bacterial isolates, limiting the significance of our conclusions. However, KPC was dominant among hospital isolates originating from Zagreb, whereas OXA-48 prevailed among community isolates. There was a small number of strains analyzed, originating mostly from the urinary tract, making correlation between resistance phenotype and sample type impossible.

There was disconcordance between the CarbaResist inter-array genotyping kit and WGS, which could be attributed to better performance of WGS which identified higher numbers of aminoglycoside- and β-lactam-resistance genes and also enables identification of the allelic variant. The CarbaResist inter-array genotyping kit is not yet recommended for diagnostic purposes. On the other hand, WGS failed to identify the *bla*_VIM_ gene in one strain. This could be due to the fact that the isolates for WGS were subcultured without the carbapenem selection pressure, which could cause plasmid loss [20,21]. *bla*_SHV_ genes from two isolates, which are chromosomal and intrinsic genes of *K. pneumoniae*, were detected by conventional PCR and WGS, but not with the CarbaResist inter-array genotyping kit. PCR reactions were performed on all 31 isolates, while the CarbaResist inter-array genotyping kit and WGS were performed only on four selected isolates due to the high cost of these tests. On the other hand, there was complete concordance between PCR and immunochromatographic test for carbapenemase detection, proving the good performance of tests which detect protein.

From the clinical point of view, FDC-resistant bacterial isolates cause “difficult-to-treat” infections. Extensive resistance profiles have severe clinical implications, since they pose challenges to both the selection of appropriate empirical and efficient targeted therapy. From a public health perspective, the remarkable ability of *K. pneumoniae* to acquire resistance to newly developed compounds, driving to the development of PDR isolates, raises concern about the risk factors, identification of the population at risk, and thus measures for the control of their spread.

In the present study, we aimed to characterize FDC-resistant isolates, in order to give insight in their resistome and molecular epidemiology. We combined phenotypic and molecular characterization of resistance traits. This is particularly important in case of carbapenemases, because sometimes they might confer only a slight increase in carbapenem MICs, as observed with the NDM producer. For this reason, a molecular approach in addition to phenotypic tests might be helpful.

MBL-producing, FDC-resistant organisms pose serious therapeutic problems, as they are resistant to novel inhibitor combinations such as ceftazidime–avibactam, ceftolozane–tazobactam and imipenem–relebactam. In addition, the presence of *arm* and *rmt* genes-encoding methylases, associated with panaminoglycoside resistance, compromise the use of aminoglycosides. In our study, resistance to FDC was coupled in the majority of cases with ESBL and carbapenemase production, and in some cases with resistance to novel β-lactam–inhibitor combinations. The best activity was demonstrated for ceftazidime–avibactam. Although colistin was susceptible in approximately two-thirds of the isolates, the monotherapy is not recommended due to development of heteroresistance and its nephrotoxicity. Aztreonam might exert good activity on MBL-positive FDC-resistant isolates, but it is not licensed for use in Croatia.

The L plasmid, an epidemic plasmid connected with the worldwide dissemination of *bla*_OXA-48_ genes, was detected in our OXA-48-producing organisms, suggesting that it could be responsible for the carbapenem resistance. The IncA/C plasmid was linked to *bla*_NDM_ genes, which is compatible with previous investigations [30], although it is not so unambiguous as with OXA-48-encoding genes as there are also other plasmids, such as IncL/M, associated with NDM [31].

The STs reported in this study had never been identified in Croatia before. In the earlier studies on CRKP, the dominant STs were ST29, ST37, ST4871 [23], ST 39, ST437 [28], ST36 and ST258 [30]. ST437 was identified in an Italian study in an FDC-resistant strain carrying the *bla*_NDM_ gene [27]. In a Croatian study, it harbored the *bla*_NDM_ and *bla*_OXA-48_ genes [28].

There are several limitations of our study. There was a small number of isolates, originating from one country. Moreover, the clarification of FDC-resistance mechanisms was not performed. STs were identified only for three isolates, and thus clonal expansion could not be ruled out. On the other hand, the strength of the study is a profound molecular analysis of the isolates, using different methodologies such as the CarbaResist inter-array genotyping kit technique and WGS.

## 4. Materials and Methods

### 4.1. Bacterial Isolates and Patients

This is a descriptive cross section study conducted in two major hospital centers and one Public Health Institute in Croatia: UHS located in southern Croatia, UHCSM and PH located in Zagreb and serving outpatient population of Zagreb. The bacterial isolates included in this study were obtained during routine microbiology testing. The bacterial collection consisted of 31 *K. pneumoniae* isolates with reduced susceptibility to FDC, collected during 2023–2024 in the participating centers. The isolates were stored at −80 °C in the glycerol-containing medium for the purpose of the study, and sent to the Clinical Department for Clinical Microbiology and Infection Prevention and Control of the University Hospital Center Zagreb for further analysis. The demographic and clinical data (age, gender, comorbidities and entire hospital courses) were retrospectively analyzed from the internet medical records, in case of hospital isolates. Species identification of the isolates was determined using MALDI-TOF MS (matrix-assisted laser desorption ionization–time of flight mass spectrometry, Bruker, Bremen, Germany), Biotyper (Bruker, Daltonik GmbH, Bremen, Germany) according to the manufacturer’s recommendations. A CarbaResist inter-array genotyping test was performed at the Institute for Hygiene, Microbiology and Environmental Medicine of the Medical University in Graz and WGS at the Institute for Technology in Vienna.

### 4.2. Antimicrobial Susceptibility Testing (AST) and Phenotypic Tests for Detection of ESBLs, Plasmid-Mediated AmpC β-Lactamases and Carbapenemases

The disk diffusion test was performed in line with EUCAST guidelines [32] in the participating centers for the purpose of routine laboratory diagnostic. If the isolate demonstrated reduced inhibition zone around FDC disk, it was subjected to further phenotypic and molecular analysis. The broth dilution method, according to CLSI (Clinical Laboratory Standard Institution) [33] was performed in order to determine the minimum inhibitory concentrations (MICs) against the wide range of antibiotics presented in Table 1. Isolates resistant to at least one carbapenem (imipenem, meropenem and ertapenem) were further tested for colistin susceptibility by the broth dilution method according to the European Committee on Antimicrobial Susceptibility Testing (EUCAST) [33]. The susceptibility to FDC, ceftazidime–avibactam, sulphamethoxazole–trimethoprim and ceftolozane–tazobactam was determined only by the disk diffusion test. Resistance to FDC was confirmed by an E-test according to the manufacturer’s recommendations. FDC resistance was defined as MIC > 2 mg/L or zone inhibition size < 23 mm [32]. The classification of the resistance phenotype of the strains was as follows: MDR strains resistant to at least three of the tested antimicrobials belonging to separate antibiotic classes; XDR strains resistant to all the tested antimicrobials except for two antimicrobial classes, PDR strains resistant to all the tested antimicrobial agents [34]. We calculated the MAR indices as described by Davis and Brown [35] according to the formula: a/b where ‘a’ was the number of antibiotics, an isolate exhibited resistance against the number of antibiotics the isolate was tested against (‘b’).

The isolates resistant to ESC were subjected to DDST for the detection of ESBLs in the participating centers as a part of a routine diagnostic practice [36]. Since the sensitivity of DDST depends on the distance between the cephalosporin disks and the central disk, the results were confirmed by combined disk test applying disks containing ESC with and without clavulanic acid, as inhibitor of class A ESBLs [34]. The screening for AmpC production was performed as described previously, considering resistance to cefoxitin as the discriminative parameter for the presence of AmpC β-lactamase [37]. The confirmation of p-AmpC positivity was carried out by DDST using cloxacillin disk as the AmpC inhibitor, placed between ceftazidime and cefotaxime disks [38]. Distortion of the inhibition zones around ESC disks towards a central disk with cloxacillin was considered a positive result [38].

Isolates with reduced susceptibility to carbapenems in the frames of routine diagnostic were subjected to immunochromatographic OKNV test which aimed to determine the carbapenemase type, important for the antibiotic choice in hospital setting [39]. Detection of carbapenemase activity released by FDC-resistant *K. pneumoniae* was carried out by MHT, according to the CLSI 2017 (M100-S31) [40]. Known carbapenemase positive and negative isolates of *K. pneumoniae* from our collection were used as quality control strains for the MHT. *E. coli* ATCC 25922 strain, susceptible to carbapenems, was cultured overnight, suspended in saline and adjusted to Mc Farland 0.5, and swabbed on MHA (Mueller–Hinton agar). Meropenem disks (10 µg) were placed in the center of Mueller–Hinton agar MHA plates, and the test isolates were streaked as a thin straight line, from the edge of the disk to the edge of the plate. The presence of the distorted inhibition zone (clover leaf-shape) of *Escherichia coli* ATCC 25922’s growth toward the meropenem disk was considered a positive result. If MHT was positive, the metal chelators (EDTA) were used to prove MBL activity, as described previously. The test was considered positive if the zone around the carbapenem disk was increased for at least 7 mm in the presence of EDTA [41].

Since the MHT was shown to lack sensitivity, alternative methods were used to confirm carbapenem hydrolysis. The mCIM and eCIM tests carried out according to the Clinical and Laboratory Standards Institute, 2021 guidelines in order to analyze the hydrolysis of the carbapenem substrate in the presence of the carbapenemase produced by the tested isolates [42]. The mCIM was performed for all the isolates, whereas the eCIM (in conjunction with mCIM) was performed in isolates that initially tested positive for mCIM.

Briefly, a heavy suspension of the test strain was prepared in brain–heart infusion broth. Subsequently, a 10-µg meropenem disk (Oxoid) was added to the suspension having the test isolate and incubated for 4 h at 35–37 °C to allow the hydrolysis of the meropenem substrate. After incubation, the disk was removed from the suspension and placed on the lawn of *E. coli* ATCC 25922, which is susceptible to meropenem and incubated overnight.

The test was interpreted as positive if the inhibition zone was less than 15 mm or there were colonies growing inside of the inhibition zone [42]. The eCIM test was performed in the same way, but with one tube containing twenty µL of 0.5 M EDTA to inhibit MBLs. The test indicated MBL positivity if there was ≥5 mm increase in zone diameter in eCIM experiment compared to the control sample without EDTA [42]. The strains from own collection, known to be positive for KPC, VIM, NDM and OXA-48, were used as positive and negative controls.

### 4.3. Molecular Detection of Resistance Genes

An in-house extraction was performed by thermal lysis. Three to five colonies were suspended in ultrapure water and lysed by heating at 95 °C for 10 min. Cellular debris was removed by centrifugation at 10,000 rpm for 2 min. All samples underwent genotypic confirmation of resistance genes by PCR assays. The isolates were screened for the presence of genes encoding broad and extended-spectrum β-lactamases (*bla*_SHV_, *bla*_TEM_, *bla*_CTX-M,_) [43,44,45] (Table 4), and fluoroquinolone resistance genes (*qnr*A, *qnr*B, *qn*rS) [46] using the primers and protocols described previously. Singleplex PCR was employed to detect *mcr-*1 and *mcr-*2 genes, among colistin-resistant isolates [47]. *bla*_SHV_ and *bla*_TEM_ PCR amplicons from six and eleven strains, respectively, were subjected to sequencing in Eurofin Genomics (https://eurofingenomics.eu, accessed on 19 December 2024). Multiplex PCR was carried out to detect the group of CTX-M β-lactamases [48], p-AmpC β-lactamase genes (Table 4) [49] and carbapenemase-encoding genes of class A, (*bla*_KPC_) class B (*bla*_VIM_, *bla*_IMP_ and *bla*_NDM_) and carbapenem-hydrolyzing oxacillinases (*bla*_OXA-48-like_) [50]. The presence of insertion sequence flanking the *bla*_CTX-M_ genes was conducted by PCR mapping with forward primer for I*SEcp1* and IS*26* combined MA-3 (reverse for *bla*_CTX-M_ genes) [29]. The genetic platform surrounding OXA-48-encoding genes was analyzed with primers for IS*1999* in combination with forward and reverse primers for *bla*_OXA-48_ [51].

### 4.4. CarbaResist Inter-Array Genotyping Kit

Genotyping of four representative *K. pneumoniae* isolates (one or two from each center) was conducted using the microarray-based CarbaResist Genotyping Kit, according to the manufacturer’s instructions, version 1012012100004 (INTER-ARRAY, fzmb GmbH, Bad Langensalza, Germany). In short, genomic DNA was isolated from monoclonal overnight cultures using the Qiagen DNeasy Blood and Tissue Kit, according to the manual. The unfragmented DNA was linear amplified using one primer for each target sequence (antisense) and internally labeled with biotin dUTP. The obtained ssDNA (single-stranded) products were transferred into the ArrayWells for hybridization. These wells contain 230 probes corresponding to distinct genes for the most relevant carbapenemases, ESBL and AmpC, as well as genes associated with β-lactam-, aminoglycoside-, fluoroquinolone-, sulphonamide-, trimethoprim- and colistin-resistance. After washing steps to remove any unbound DNA, horseradish peroxidase (HRP)-conjugated streptavidin was bounded to all hybridized sections, resulting in dark spots on the chip due to an enzymatic reaction. The detection of these spots and data acquisition was performed automatically using the INTER-VISION Reader.

### 4.5. Whole Genome Sequencing (WGS)

The genomes of the four representative isolates were sequenced using next generation sequencing [52]. First, the strains were cultivated in tryptic soy broth (TSB) (Merck Millipore, MA, USA) at 37 °C overnight. Then, the genomic DNA was extracted using the QIAamp DNA Mini Kit (Qiagen, Hilden, Germany) according to the manufacturer’s instructions. The DNA extracts were sent to the Next Generation Sequencing Facility of the Vienna Biocenter for sequencing using the Oxford Nanopore Technology (ONT) MinION system, according to the manufacturer’s instructions. The single reads obtained were assembled and analyzed using the commercial software platform Solu from Solugenomics (https://www.solugenomics.com/ accessed on 18 December 2024). The platform has integrated Dragonflye (Version 1.2.1) to specifically assemble ONT reads. The assembled sequences were of very high quality, resulting in only 5, 6, 13, and 25 contigs, respectively, (UG 65,815: N50 5,402,933; UG 76,341: N50 5,322,187; VG-34989: N50 1,360,740; 8086-24: N50 865,205). They were further analyzed using the webservers and services of the Center for Genomic Epidemiology (http://www.genomicepidemiology.org (accessed on 24 October 2024)) [52].

The sequences were deposited in the NCBI Gen Bank, and the accession numbers are provided in Table 3.

### 4.6. Characterization of Plasmids

PCR-based replicon typing (PBRT) [53] was applied for the molecular typing of plasmids associated with resistance genes among Enterobacterales. The procedure was performed with eighteen pairs of primers including five multiplex and three singleplex PCR which reveal the plasmid incompatibility group (Inc.). An updated method was used for the IncL plasmid, which usually carries *bla*_OXA-48_ genes [54].

### 4.7. Genotyping of the Isolates by Multilocus Sequence Typing (MLST)

MLST was applied on two representative *K. pneumoniae* isolates (1-(UG65815 and 4-(8086-24) by amplifying seven housekeeping genes (*gap*, *pho*, *pgi*, *inf*, *ton*B, *rpo*B, *mdh*) according to the protocol of Diancourt et al. [55] For one strain (3-VG-34989), ST was retrieved from WGS. Sequence analysis of PCR amplicons was carried out by Eurofins Genomics (https://eurofingenomics.eu, accessed on 19 December 2024).

## 5. Conclusions

To our knowledge, this is the first study on FDC resistance in Croatia. The diffusion of FDR-resistant isolates was detected in both hospital and outpatient settings, emphasizing the need for One health approach. Croatia is one of the countries with a high rate of antibiotic resistance, and where antibiotics are used excessively and often inappropriately, resulting in a high rate of carbapenem resistance (19%) according to EARS data [18]. The fact that FDC resistance was coupled with carbapenemase and ESBL production, and, in some cases with colistin resistance, left only a few or no therapeutic options available. The emergence and spread of this dangerous superbug raises concern and call for a change in public health policy regarding the use of antibiotics. New β-lactam antibiotics and FDC remain an important addition to the antibiotic armamentarium, but their use must be constantly monitored to avoid the rapid development of resistance. Although they have shown great promise, experience with their use is still limited.

## Figures and Tables

**Table 1:** Antibiotic susceptibility, phenotypic tests for carbapenemases, and β-lactamase content of FDC-resistant *K. pneumoniae* isolates.

	Center	Strain	Resistance Phenotype	Hodge/ CIM/eCIM	β-Lactamase Content
1	UHS	UG65815	AMC, TZP, CAZ, CTX, CRO, FEP, FDC, IMI, MEM, ERT, GM, CIP, CZA, C/T	+/+/+	VIM-1, NDM-5, CTX-M SHV
2	UHS	UG76341	AMC, TZP, CAZ, CTX, CRO, FEP, FDC, IMI, MEM, ERT, GM, AMI, CIP, CZA, C/T	+/+/+	OXA-48, NDM, CTX-M, SHV
3	UHS	UG72466	AMC, TZP, CAZ, CTX, CRO, FEP, FDC, IMI, MEM, ERT, CIP, C/T	+/+/−	OXA-48, CTX-M, SHV-1
4	UHS	UG54341	AMC, TZP, CAZ, CTX, CRO, FEP, FDC, ERT, GM, AMI, CIP, CZA, C/T	+/+/−	NDM, SHV
5	UHS	UG68640	AMC, TZP, CAZ, CTX, CRO, FEP, FDC, IMI, MEM, ERT, GM, AMI, CIP, CZA, C/T	+/+/−	OXA-48, CTX-M, SHV, TEM-1
6	UHS	UG72747	AMC, TZP, CAZ, CTX, CRO, FEP, FDC, IMI, MEM, ERT, GM, AMI, CIP, COL, C/T	+/+/−	OXA-48, CTX-M, SHV
7	UHS	UG78315	AMC, TZP, CAZ, CTX, CRO, FEP, FDC, IMI, MEM, ERT, GM, AMI, CIP, C/T	+/+/−	OXA-48,, CTX-M, SHV
8	UHS	UG85877	AMC, TZP, CAZ, CTX, CRO, FEP, FDC, IMI, MEM, ERT, GM, AMI, CIP, C/T	+/+/−	OXA-48, CTX-M, SHV
9	UHS	UG78871	AMC, TZP, CAZ, CTX, CRO, FEP, FDC, IMI, MEM, ERT, GM, AMI, CIP, COL, C/T	−/−	OXA-48, CTX-M, SHV
10	UHS	UG81973	AMC, TZP, CAZ, CTX, CRO, FEP, FDC, IMI, MEM, ERT, GM, AMI, CIP, COL, C/T	−/−	OXA-48, CTX-M, SHV
11	UHS	UG45741	AMC, TZP, CAZ, CTX, CRO, FEP, FDC, IMI, MEM, ERT, GM, AMI, CIP, C/T	+/+/−	OXA-48, CTX-M, SHV
12	UHS	UG78464	AMC, TZP, CAZ, CTX, CRO, FEP, FDC, IMI, MEM, ERT, GM, AMI, CIP, COL, C/T	+/−	OXA-48, CTX-M, SHV-1
13	UHS	UG75475	AMC, TZP, CAZ, CTX, CRO, FEP, FDC, IMI, MEM, ERT, GM, AMI, CIP, C/T	+/−	OXA-48, CTX-M, SHV-1, TEM-1
14	UHCSM	VG34989	AMC, TZP, CAZ, CTX, CRO, FEP, FDC, IMI, MEM, ERT, GM, AMI, CIP, COL, C/T	+/+/−	OXA-48, CTX-M, SHV-1, TEM-1
15	UHCSM	VG51854	AMC, TZP, CAZ, CTX, CRO, FEP, FDC, IMI, MEM, ERT, GM, AMI, CIP, C/T	+/+/−	KPC, TEM-1, SHV
16	UHCSM	VG51612	AMC, TZP, CAZ, CTX, CRO, FEP, FDC, IMI, MEM, ERT, GM, AMI, CIP, C/T	+/+/−	KPC, TEM-1, SHV
17	UHCSM	VG51788	AMC, TZP, CAZ, CTX, CRO, FEP, FDC, IMI, MEM, ERT, GM, AMI, CIP, C/T	+/+/−	KPC, TEM-1, SHV-11
18	UHCSM	VG52055	AMC, TZP, CAZ, CTX, CRO, FEP, FDC, IMI, MEM, ERT, GM, AMI, CIP, C/T	+/+/−	KPC, TEM-1, SHV-1
19	UHCSM	VG54301	AMC, TZP, CAZ, CTX, CRO, FEP, FDC, IMI, MEM, ERT, GM, AMI, CIP, COL, C/T	+/+/−	KPC, TEM-1, SHV,
20	UHCSM	VG56379	AMC, TZP, CAZ, CTX, CRO, FEP, FDC, IMI, MEM, ERT, GM, AMI, CIP, C/T	+/+/+	KPC, TEM-1, SHV
21	PH	80862-24	AMC, TZP, CAZ, CTX, CRO, FEP, FDC, IMI, MEM, ERT, GM, AMI, CIP, COL, CZA, C/T	+/+/+	OXA-48+NDM
22	PH	51785-24	AMC, TZP, CAZ, CTX, CRO, FEP, FDC, IMI, MEM, ERT, GM, AMI, CIP, C/T	+/+/−	KPC, TEM, SHV, TEM-1
23	PH	46551-24	AMC, TZP, CAZ, CTX, CRO, FEP, FDC, IMI, MEM, ERT, GM, AMI, CIP, C/T	+/+/−	OXA-48, CTX-M, SHV
24	PH	45896-24	AMC, TZP, CAZ, CTX, CRO, FEP, FDC, IMI, MEM, ERT, GM, AMI, CIP, C/T	+/+/−	OXA-48, CTX-M, SHV, TEM-1
25	PH	49359-24	AMC, TZP, CAZ, CTX, CRO, FEP, FDC, IMI, MEM, ERT, GM, AMI, CIP, C/T	+/+/−	OXA-48, CTX-M, SHV
26	PH	46238-24	AMC, TZP, CAZ, CTX, CRO, FEP, FDC, IMI, MEM, ERT, GM, AMI, CIP, COL, C/T	+/+/−	OXA-48, CTX-M, SHV, TEM
27	PH	51750-24	AMC, TZP, CAZ, CTX, CRO, FEP, FDC, IMI, MEM, ERT, GM, AMI, CIP, C/T	+/+/−	KPC, TEM-1, SHV
28	PH	46092-24	AMC, TZP, CAZ, CTX, CRO, FEP, FDC, IMI, MEM, ERT, GM, AMI, CIP, C/T	+/+/−	KPC, TEM-1, SHV
29	PH	56620-24	AMC, TZP, CAZ, CTX, CRO, FEP, FDC, IMI, MEM, ERT, GM, AMI, CIP, COL, C/T	+/+/−	OXA-48, CTX-M, SHV
30	PH	53807-24	AMC, TZP, CAZ, CTX, CRO, FEP, FDC, IMI, MEM, ERT, GM, AMI, CIP, C/T	+/+/−	OXA-48, CTX-M, SHV
31	PH	51785-24	AMC, TZP, CAZ, CTX, CRO, FEP, FDC, IMI, MEM, ERT, GM, AMI, CIP, C/T	+/+/−	KPC, TEM, SHV

Abbreviations: AMC—amoxycillin/clavulanic acid; TZP—piperacillin–tazobactam; CAZ—ceftazidime; CRO—ceftriaxone; FEP—cefepime; IMI—imipenem; MEM—meropenem; GM—gentamicin; AMI—amikacin; CIP—ciprofloxacin; COL—colistin; C/T—ceftolozane–tazobactam; CZA—ceftazidime–avibactam.

**Table 2:** Analysis of four representative *K. pneumoniae* isolates’ antibiotic resistance genes by the CarbaResist inter-array genotyping kit.

Isolate	β-Lactam	Aminoglycosides	Fluoroquinolones	Sulphonamides	Trimethoprim	Efflux Pump
1 (UG65815)	*bla*_VIM_ *bla*_NDM_ *bla*_oxa-1_	*aac(6’)-Ib* *aadA1* *aadA2*	*qnrB*	*Sul*1	*dfrA12*	
2 (UG76341)	*bla*_NDM_ IS*Ecp-bla*_CTX-M-15_ *bla*_TEM_ *bla*_oxa-1_	*aac(6’)-Ib* *aphA*		*Sul*1		*oqxA*
3 (VG-34989)	*bla*_OXA-48_ IS*Ecp-bla*_CTX-M-15_ *bla*_SHV_	*aadA2* *armA*		*Sul*1		*oqxA* *oqxB*
4 (8086-24)	*bla*_NDM_ *bla*_OXA-48_ IS*Ecp-bla*_CTX-M-15_ *bla*_SHV_	*aadA2* *rmtC*		*Sul*1		*oqxB*

**Table 3:** WGS of four representative FDC-resistant *K. pneumoniae* isolates. Resistance genes for each antibiotic class are shown and the accession number is provided in the parentheses.

Isolate	β-Lactam	Aminoglycosides	Sulphonamide	Trimethoprim	Chloramphenicol	Efflux Pumps	Plasmid Inc. Group
1 (UG65815)	*bla*_NDM-5_ *bla*_OXA-1_ *bla*_SHV-187_	*Aph(3)-VI* (APPJ01000012) *Aph(3″)Ib* (AF321550) *aadA2* (JQ364967) *aac/6″)-Ib-cr* (HQ170510)	*Sul*1 (EU780013)	*dfrA12* (AM040708)	*catB3* (U13880)		Col(pHAD28) (KU674895 ColpVC (JX133088) IncFIB(K) (JN233704) IncN (AY046276) IncR (DQ449578) IncX3 (JN247852)
2 (UG 76341)	*bla*_NDM-1_ (FN396876) *bla*_CTX-M-15_ (AY044436) *bla*_TEM-1B_ (AY458016) *bla*_OXA-1_ (HQ1705109) *bla*_SHV-28_ (AF299299)	*aac(6″)-Ib-cr* (DQ303918) *aac(3″)-Ia* (V00359) *aphA* (M28829)	*Sul*1 (EU780013) *Sul*2 (AY034138)			*Oqx*B (EU370913)	ColRNAI (DQ298019) IncFIB(K) (JN233704) IncFII(K) (CP000648) IncL (JN626286)
3 (VG-34989)	*bla*_NDM-5_ (JN104597) *bla*_OXA-48_ (AY236073) *bla*_CTX-M-15_ (AY044436) *bla*_SHV-158_ (JX121125)	*aadA2* (JQ364967) *armA* (AY220558)	*Sul*1 (U12338)	*dfrA12* (AM040708)		*Oqx*B (EU370913)	IncFIB (JN233705) IncL (JN626286) IncX3 (JN247852)
4 (8086-24)	*bla*_NDM-5_ (FN396876) *bla*_OXA-48_ (AY236073) *bla*_CTX-M-15_ (AY044436) *bla*_SHV-158_ (JX121125)	*aadA2* (D43625) *rmtC* (AB194779)	*Sul*1 (U12338)			*Oqx*B (EU370913)	IncFII (CP000670) IncL (JN626286)

**Table 4:** Primers used in the study. Annealing temperature and the product length is provided.

Gene	Primer Sequence 5′-3′	Annealing Temperature	Product Length	Reference
*bla*_TEM_	5′-ATG-AGT-ATT-CAA-CAT-TTC-CG-3′ 5′-CCA-ATG-CTT-AAT-CAG-TGA-GG-3′	55	850	[43]
*bla*_SHV_	5′-TTC-GCC-TGT-GTA-TTA-TCT-CCC-3′ 5′-TTA-GCG-TTG-CCA-GTG-YTC-GAT-3′	58	1000	[44]
*bla*_CTX-M_	5′-SCS-ATG-TGC-AGY-ACC-AGT-AA-3′ 5′-CGC-CRA-TAT-GRT-TGG-TGG-TG-3′	55	550	[45]
*bla*_CTX-M-1_	5′-AAA-AAT-CAC-TGC-GCC-AGT--TC-3′ 5′-TTG-GTG-ACG-ATT-TTA-GCC-GC-3′	52	415	[48]
*bla*_CTX-M-2_	5′-CGA-CGC-TAC-CCC-TGC-TAT-T--3′ 5′-CCA-GCG-TCA-GAT-TTT-TCA-GG-3′	52	552	[48]
*bla*_CTX-M-9_	5′-CAA-AGA-GAG-TGC-AAC-GGA-TG-3′3′ 5′ATT-GGA-AAG-CGT-TCA-TCA-CC-3′	52	205	[48]
*bla*_CTX-M-8_	5′-TCG-CGT-TAA-GCG-GAT-GAT-GC-3′ 5′-AAC-CCA-CGA-TGT-GGG-TAG-C-3′	52	666	[48]
*bla*_CTX-M-25_	5′-GCA-CGA-TGA-CAT-TCG-GG-3′ 5′-AAC-CCA-CGA-TGT-GGG-TAG-C-3′	52	327	[48]
*bla*_MOX_	5′GCT-GCT-CAA-GGA-GCA-CAG-GAT-3′ 5′CAC-ATT-GAC-ATA-GGT-GTG-GTG-C-3′	64	520	[49]
*bla*_CMY_	5′TGG-CCA-GAA-CTG-ACA-GGC-AAA-3′ 5′TTT-CTC-CTG-AAC-GTG-GCT-GGT-3′	64	462	[49]
*bla*_DHA_	5′AAC-TTT-CAC-AGG-TGT-GCT-GGG-T-3′ 5′CCG-TAC-GCA-TAC-TGG-CTT-TGC-3′	64	405	[49]
*bla*_ACC_	5′AAC-AGC-CTC-AGC-AGC-CGG-TTA-3′ TTC-GCC-GCA-ATC-ATC-CCT-AG-3′	64	346	[49]
*bla*_MIR_	5′TCG-GTA-AAG-CCG-ATG-TTG-CGG 5′CTT-CCA-CTG-CGG-CTG-CCA-GTT-3′	64	302	[49]
*bla*_FOX_	5′AAC-ATG-GGG-TAT-CAG-GGA-GAT-G-3′ 5′CAA-AGC-GCG-TAA-CCG-GAT-TGG-3′	64	190	[49]
*bla*_IMP_	5′GGAATAGAGTGGCTTAAYTCTC-3′ GGTTTAAYAAAACAACCACC-3′	52	232	[50]
*bla*_VIM_	5-’GATGGTGTTTGGTCGCATA-3′ 5-’CGAATGCGCAGCACCAG-3′	52	390	[50]
*bla*_NDM_	5′-GGTTTGGCGATCTGGTTTTC-3′ 5′-CGGAATGGCTCATCACGATC-3′	52	621	[50]
*bla*_KPC_	5′CGTCTAGTTCTGCTGTCTTG-3′ 5′-CTTGTCATCCTTGTTAGGCG-3′	52	798	[50]
*bla*_OXA-48_	5′-GCGTGGTTAAGGATGAACAC-3′ 5′-CATCAAGTTCAACCCAACCG-3′	52	438	[50]

## Data Availability

The original contributions presented in the study are included in the article, further inquiries can be directed to the corresponding author.

## Supplementary Materials

The following supporting information can be downloaded at: https://www.mdpi.com/article/10.3390/antibiotics14020154/s1, Table S1: Minimum inhibitory concentrations (MICs) of antibiotics against FDC-resistant *K. pneumoniae* isolates. Resistance breakpoint is shown below the antibiotic abbreviation.

## Abbreviations

The following abbreviations are used in this manuscript:

AMC—amoxycillin/clavulanic acid; TZP—piperacillin–tazobactam; CXM—cefuroxime; CAZ—ceftazidime; CTX—cefotaxime; CRO—ceftriaxone; FEP—cefepime; IMI—imipenem; MEM—meropenem; GM—gentamicin; AMI—amikacin; CIP—ciprofloxacin; COL—colistin; C/T—ceftolozane–tazobactam; CZA—ceftazidime–avibactam; FDC—cefiderocol; MHT—modified Hodge test; CIM—carbapenem inactivation method; eCIM—EDTA-CIM test; ESC—expanded-spectrum cephalosporins; MIC—minimum inhibitory concentration; DDST—double disk synergy test; MBL—metallo-β-lactamase; MARI—multiple antibiotic resistance indices; CHDL—carbapenem-hydrolyzing oxacillinase; CRKP—carbapenem-resistant Klebsiella pneumoniae; UHS—University Hospital Split; UHCSM—University Hospital Center Sestre Milosrdnice; PH—Dr Andrija Štampar Public Health Institute; MHA—Mueller–Hinton agar; ESBL—extended-spectrum β-lactamase; p-AmpC—plasmid-mediated AmpC β-lactamase; ST—sequence type
