# Supplementary material for: Characterization of Klebsiella pneumoniae Isolates Resistant to Cefiderocol from Hospitals and Outpatient Settings in Croatia
Source: Antibiotics (Basel). 2025 Feb 4;14(2):154. doi: 10.3390/antibiotics14020154 (PMC11851357; doi:10.3390/antibiotics14020154)
Supplement: Supplementary file 1 [file antibiotics-14-00154-s001.zip › antibiotics-3423161-supplementary.pdf]

antibiotics

MDPI

Supplementary Materials

Characterization of *Klebsiella pneumoniae* Isolates Resistant to Cefiderocol from Hospitals and Outpatient Settings in Croatia

Table S1. Antibiotic susceptibility and β-lactamase content of cefiderocol-resistant *K. pneumoniae* isolates.

			Minimum Inhibitory Concentration (MIC)																	
	Center	Protocol number	AMC (≥32/16)	TZP ≥(128/4)	CXM (≥32)	CAZ (≥16)	CTX (4)	CRO (≥4)	FEP (≥16)	FDC (>2)	IMI (≥4)	MEM (≥4))	GM (≥16)	AMI (≥64)	CIP (≥0,25)	COL (>2)	CZA	C/T	Hodge/ CIM/eCIM/	β- lac-tamase content
1	UHCS	UG65815	>128/16(R)	>128(R)	>128(R)	>128(R)	>128(R)	>128(R)	>128(R)	>128(R)	>128(R)	>128(R)	>128(R)	>128(R)	>128(R)	0,5(S)	R	R	+/+/+	VIM-1, NDM-5, CTX-M SHV
2	UHCS	UG 76341	>128/16(R)	>128(R)	>128(R)	>128(R)	>128(R)	>128(R)	>128(R)	>128(R)	>128(R)	>128(R)	>128(R)	>128 (R)	>128 (R)	0,5(S)	R	R	+/+/+	OXA-48+NDM, CTX-M, SHV
3	UHCS	UG 72466	>128/16(R)	128(R)	>128(R)	32(R)	>128(R)	>128(R)	64(R)	8(R)	8(R)	32(R)	1(S)	2(S)	>128(R)	1(S)	S	R	+/+/-	OXA-48, CTX-M, SHV
4	UHCS	UG 54341	>128/16(R)	128(R)	>128(R)	>128(R)	>128(R)	>128(R)	128(R)	3(R)	1(R)	2(R)	>128(R)	>128(R)	>128(R)	1(S)	R	R	+/+/-	NDM, SHV, TEM
5	UHCS	UG 68640	>128/16(R)	128(R)	>128(R)	16(R)	>128(R)	>128(R)	16(R)	4(R)	64(R)	>128(R)	>128(R)	>128(R)	>128(R)	1(S)	S	R	+/+/-	OXA-48, CTX-M, SHV
6	UHCS	UG 72747	>128/16(R)	128(R)	>128(R)	128(R)	>128(R)	>128(R)	32(R)	4(R)	64(R)	>128(R)	>128(R)	>128(R)	>128(R)	64(R)	S	R	+/+/-	OXA-48, CTX-M, SHV
7	UHCS	UG78315	>128/16(R)	128(R)	>128(R)	32(R)	>128(R)	>128(R)	>128(R)	8(R)	16(R)	32(R)	128(R)	>128(R)	>128(R)	1 (S)	S	R	+/+/-	OXA-48,, CTX-M, SHV

8	UHCS	UG 85877	>128/16(R)	128(R)	>128(R)	32(R)	>128(R)	>128(R)	>128(R)	8(R)	32(R)	64(R)	128(R)	>128(R)	>128(R)	0,5 S)	S	R	+/+/-	OXA-48, CTX-M, SHV
9	UHCS	UG78871	>128/16(R)	128(R)	>128(R)	32(R)	>128(R)	>128(R)	>128(R)	8(R)	32(R)	64(R)	>128(R)	>128(R)	>128(R)	128(S)	S	R	-/-	OXA-48, CTX-M, SHV
10	UHCS	UG81973	>128(R)	>128(R)	>128(R)	32(R)	>128(R)	>128(R)	>128(R)	8(R)	64(R)	64(R)	>128(R)	>128(R)	>128(R)	128(R)	S	R	-/-	OXA-48, CTX-M, SHV
11	UHCS	UG45741	>128(R)	128(R)	>128(R)	64(R)	>128(R)	>128(R)	>128(R)	8(R)	16(R)	64(R)	>128(R)	>128(R)	>128(R)	0,5(S)	S	R	+/+/-	OXA-48, CTX-M, SHV
12	UHCS	UG 78464	>128(R)	128(R)	>128(R)	>128(R)	>128(R)	>128(R)	>128(R)	8(R)	32(R)	64(R)	>128(R)	>128(R)	>128(R)	32(R)	S	R	+/-	OXA-48, CTX-M, SHV
13	UHCS	UG75475	>128(R)	128(R)	>128(R)	16(R)	>128(R)	>128(R)	>128(R)	8(R)	32(R)	32(R)	>128(R)	>128(R)	>128(R)	2 (S)	S	R	+/-	OXA-48, CTX-M, SHV
14	UHCSM	VG34989	>128(R)	128(R)	>128(R)	>128(R)	>128(R)	>128(R)	32(R)	8(R)	4(R)	4(R)	64(R)	16 (S)	>128(R)	16(R)	S	R	+/+/-	OXA-48, CTX-M, SHV
15	UHCSM	VG51854	>128(R)	128(R)	>128(R)	64(R)	>128(R)	>128(R)	64(R)	16(R)	32(R)	64(R)	>128(R)	>128(R)	>128(R)	0,5 (S)	S	R	+/+/-	KPC, TEM, SHV, TEM
16	UHCSM	VG51612	>128(R)	128(R)	>128(R)	64(R)	>128(R)	>128(R)	32(R)	4(R)	64(R)	128(R)	64(R)	64(R)	>128(R)	0,5 (S)	S	R	+/+/-	KPC, TEM, SHV
17	UHCSM	VG51788	>128(R)	128(R)	>128(R)	128(R)	>128(R)	>128(R)	64(R)	4(R)	32(R)	64(R)	>128(R)	>128(R)	>128(R)	0,5 (S)	S	R	+/+/-	KPC, TEM, SHV
18	UHCSM	VG 52055	>128(R)	>128(R)	>128(R)	>128(R)	>128(R)	>128(R)	64(R)	8(R)	16(R)	8(R)	>128(R)	>128(R)	>128(R)	0,5 (S)	S	R	+/+/-	KPC, TEM, SHV
19	UHCSM	VG54301	>128(R)	>128(R)	>128(R)	>128(R)	>128(R)	>128(R)	>128(R)	8(R)	32(R)	64(R)	>128(R)	>128(R)	>128(R)	8	S	R	+/+/-	KPC, TEM, SHV,
20	UHCSM	VG56379	>128(R)	>128(R)	>128(R)	>128(R)	>128(R)	>128(R)	>128(R)	8(R)	64(R)	>128(R)	>128(R)	>128(R)	>128(R)	0,5 (S)	S	R	+/+/+	KPC, TEM, SHV
21	PH	8086/-2-24	>128(R)	>128(R)	>128(R)	>128(R)	>128(R)	>128(R)	>128(R)	8(R)	64(R)	128(R)	>128(R)	>128(R)	>128(R)	32(R)	R	R	+/+/+	OXA-48+NDM
22	PH	51785-2-24	>128(R)	128(R)	>128(R)	32(R)	>128(R)	128(R)	32(R)	8(R)	128(R)	128(R)	64(R)	32 (R)	>128(R)	0,5 (S)	S	R	+/+/-	KPC, TEM, SHV, TEM

23	PH	46551-2-24	>128(R)	>128(R)	>128(R)	32(R)	>128(R)	>128(R)	32(R)	8(R)	8(R)	32(R)	>128(R)	>128(R)	>128(R)	0,5 (S)	S	R	+/+/-	OXA-48, CTX-M, SHV
24	PH	45896-2-24	128(R)	128(R)	>128(R)	32(R)	64(R)	16(R)	16(R)	8(R)	16(R)	32(R)	64(R)	32(R)	>128(R)	0,5 (S)	S	R	+/+/-	OXA-48, CTX-M, SHV, TEM
25	PH	49359-2-24	>128(R)	>128(R)	>128(R)	64(R)	>128(R)	>128(R)	64(R)	4(R)	8(R)	32(R)	128(R)	32(R)	>128(R)	0,5 (S)	S	R	+/+/-	OXA-48, CTX-M, SHV
26	PH	46238-2-24	>128(R)	>128(R)	>128(R)	16(R)	>128(R)	>128(R)	32(R)	8(R)	8(R)	32(R)	>128(R)	>128(R)	>128(R)	16(R)	S	R	+/+/-	OXA-48, CTX-M, SHV, TEM
27	PH	51750-2-24	>128(R)	>128(R)	>128(R)	64(R)	>128(R)	>128(R)	32(R)	8(R)	>128(R)	>128(R)	>128(R)	>128(R)	>128(R)	0,5 (S)	S	R	+/+/-	KPC, TEM, SHV
28	PH	46092-2-24	>128(R)	>128(R)	>128(R)	64(R)	>128(R)	>128(R)	32(R)	8(R)	>128(R)	>128(R)	>128(R)	>128(R)	>128(R)	0,5 (S)	S	R	+/+/-	KPC, TEM, SHV
29	PH	56620/2-24	>128(R)	>128(R)	>128(R)	128(R)	>128(R)	>128(R)	32(R)	4(R)	8(R)	16(R)	>128(R)	>128(R)	>128(R)	8(R)	S	R	+/+/-	OXA-48, CTX-M, SHV
30	PH	53807/2-24	>128(R)	>128(R)	>128(R)	128(R)	>128(R)	>128(R)	64(R)	4(R)	8(R)	16(R)	>128(R)	>128(R)	>128(R)	0,5(S)	S	R	+/+/-	OXA-48, CTX-M, SHV
31	PH	51785/2-24	>128 (R)	128 (R)	>128 R)	32 (R)	>128 R)	>128(R))	>128(R))	4(R)	>128(R)	>128(R)	>128(R)	>128(R)	>128(R)	1(S)	S	R	+/+/-	KPC, TEM, SHV

Abbreviations: AMC—amoxycillin–clavulanic acid; TZP—piperacillin–tazobactam; CXM—cefuroxime; CAZ—ceftazidime; CTX—cefotaxime; CRO—ceftriaxone; FEP—cefepime; IMI—imipenem; MEM—meropenem; GM—gentamicin; AMI—amikacin; CIP—ciprofloxacin; COL—colistin, C/T—ceftolozane–tazobactam; CZA—ceftazidime–avibactam; CIM—carbapenem inactivation method; eCIM—EDTA-CIM test; UHCSM—University Hospital Sestre Milosrdnice; UHS—University Hospital Centre Split; PH—Dr. Andrija Štampar Teaching Public Health Institute. Resistance breakpoint is provided below the antibiotic abbreviation: R—resistance; S—susceptibl
